# Keeping distance or getting closer: How others’ emotions shape approach-avoidance postural behaviors and preferred interpersonal distance

**DOI:** 10.1371/journal.pone.0298069

**Published:** 2024-02-02

**Authors:** Angélique Lebert, Dorine Vergilino-Perez, Laurence Chaby

**Affiliations:** 1 Université Paris Cité, Vision Action Cognition, Boulogne-Billancourt, France; 2 Department of Psychiatry and Forensic Medicine, Autonomous University of Barcelona, Cerdanyola del Valles, Barcelona, Spain; 3 Sorbonne Université, CNRS, Institut des Systèmes Intelligents et de Robotique (ISIR), Paris, France; 4 Université Paris Cité, Boulogne-Billancourt, France; Universidad Complutense Madrid, SPAIN

## Abstract

Understanding the influence of emotions on social interactions is important for a global understanding of the dynamics of human behavior. In this study, we investigated the interplay between emotions, spontaneous approach or avoidance tendencies, and the regulation of interpersonal distance. Fifty-seven healthy adults participated in a three-part experiment involving exposure to approaching or withdrawing emotional faces (neutral, happy, sad, fearful, disgusted, angry). The sequence began with an initial computerized stop-distance task, followed by a postural task in which participants’ approach or avoidance tendencies were quantified via center of pressure (CoP-Y) displacements on a force platform, and concluded with a final computerized stop-distance task. Our findings revealed a gradient in postural responses, with the most forward CoP-Y displacements for neutral and happy faces, indicative of approach tendencies. These were followed by lesser forward displacements for sad and fearful faces, and most pronounced backward displacements for disgusted and angry faces, indicating avoidance. Furthermore, we observed modulations in participants’ preferred interpersonal distance based on emotional cues, with neutral and happy faces associated with shorter distances, and disgusted and angry faces linked to larger distances. Despite these similar results, no direct correlation was found between CoP-Y and preferred interpersonal distance, underscoring a dissociation between spontaneous and voluntary social behaviors. These results contribute to a better understanding of how emotional expressions shape social interactions and underscore the importance of considering emotional cues, postural action tendencies, and interpersonal distance in facilitating successful social interactions.

## Introduction

In human social interactions, the body and the space immediately surrounding it are of utmost importance [1]. Personal space is defined as the area around the body where intrusion can cause feelings of discomfort [2, 3] or even threats [4], especially when the distance to others is approximately 100 cm and below [5]. This potential discomfort triggers physiological arousal [6–9] and may result in bodily behaviors that evoke fight, flight, or immobilization responses [10], leading individuals to regulate their social distance from others [11]. However, it remains unclear whether this social distance for interacting with others differs from more spontaneous bodily behaviors, such as approach or avoidance tendencies.

Interpersonal distance can be considered as a nonverbal form of communication [12] which can be dynamically regulated depending on whether an encounter represents a threat or an opportunity for social engagement. Such regulations rely mainly on nonverbal emotional signals from others [13], including gaze [6, 14, 15] and facial expressions [16–19]. This is primarily due to the adaptive [20, 21] and motivational [22, 23] values of emotions, shaping individuals’ relationships with the world and enabling action. Traditionally, the motivational theory of emotions has suggested that emotional stimuli automatically elicit behavioral reactions—positive stimuli leading to approach behaviors and negative initiating avoidance [23, 24]. However, this straightforward relationship is increasingly being questioned, and it is still unclear whether anger and/or fear prime an approach or avoidance behavior, challenging the traditional framework [25–27]. Furthermore, recent studies indicate that the impact of emotions on behavior may be contingent on the relevance of the task to the participants’ goals [28–30], or on individual characteristics such as personality traits [31, 32]). Finally, it has been shown that emotions perceived in others can also influence behavior based on the potential for future interactions. This can manifest in affiliative tendencies, like the desire to approach someone pleasant [33]. However, such responses are not limited to positive emotions. Contrary to intuitive assumptions, expressions of fear or sadness can also elicit prosocial behaviors and be interpreted as cues for social engagement and connection. It has been illustrated that fear, while potentially indicating a threat, can function as an affiliative stimulus leading to approach behaviors like helping [34]. Similarly, it has been highlighted that perceptions of emotional distress can trigger supportive actions like comforting someone who seems sad [35].

The assessment of preferred interpersonal distance is a crucial component of the study of social behavior. In laboratory settings, tasks have been developed to allow participants to adjust their distance from others based on their level of comfort. One commonly used paradigm is the ‘stop-distance’ task, which involves stopping an approaching partner at the point where they still feel comfortable with the other’s proximity [36–38]. Other measures have also been employed to assess preferred interpersonal distance, including paper-and-pencil methods [39–41] and computer-based or virtual reality tasks, in which participants had to voluntarily adjust their preferred distance in response to stimuli representing a virtual partner [5, 42–44]. However, computer-based or virtual reality tasks offer the possibility of manipulating partner characteristics such as age, gender or facial expression, while offering better experimental control and replicability [45]. This makes them valuable tools for investigating interpersonal distance adjustments in diverse situations.

In social contexts, facial expressions serve as social cues [46, 47], providing valuable information about the emotional states of others, and facilitating interpersonal communication by adjusting and coordinating with them [48]. Studies have shown that emotional facial expressions can influence both estimation and adjustment of interpersonal distance, with emotional expressions judged closer than neutral expressions [17]. Positive and negative facial expressions have also been found to affect judgments of proximity, with friendly faces or faces with happy or neutral expressions leading to shorter preferred interpersonal distances, and negative expressions, such as anger leading to larger distances [18, 19, 44]. Note that most of these studies only explored a limited range of emotions, usually two or three, and have frequently overlooked basic emotions such as disgust, which can be perceived as a threat, particularly in situations involving contamination or illness [49]. Interestingly, in the era of COVID-19, recent studies have examined how wearing face masks —by reducing the probability of contamination and enhancing trustworthiness— can offer a more nuanced perspective on our understanding of interpersonal distance regulation [50, 51]. In this context, Scerrati et al. (2022) [52] highlighted the influence of the pandemic on individuals’ assessment of social proximity.

Although individuals generally feel uneasy in close proximity to strangers, they are more likely to approach someone who appears happy or in distress, while avoiding those who appear angry [53, 54]. When faced with potential threats, individuals may also exhibit freezing behavior, characterized by reduced body motion and increased muscles tension [55, 56], which is thought to facilitate perceptual and attentional processes aimed at triggering appropriate actions [24, 57]. The inclination to seek positive social interactions and avoid potentially negative ones may be explained by theoretical frameworks that describe action tendencies and approach-avoidance behaviors in humans [58, 59]. Thus, evaluation of the emotional situation can lead to action tendencies that are not necessarily overt actions, but rather readiness or preparation for action [23, 60, 61]. These tendencies may manifest in bodily mobilization and posture, aimed at achieving a more favorable or less unfavorable situation for the individual [62, 63].

Following this logic, several authors have suggested that body movements are a direct and ecologically valid way to study action tendencies related to approach and avoidance behaviors. Classical approaches have utilized manual tasks in which participants are instructed to either pull a lever or a joystick toward themselves (i.e., approach) or push it away from themselves (i.e., avoidance) in response to different emotional stimuli [64, 65]. More recent studies have examined whole-body changes by instructing participants to step toward or move away from a screen showing emotional facial expressions [66, 67], while others have focused on more implicit changes reflected in postural shifts when participants are in quiet standing [68, 69]. Incorporating posturography in studies of approach-avoidance behaviors is useful, as it provides insights into the underlying mechanisms of these behaviors and their relationship with emotional processing. Posturography allows recording and quantifying small body sway reflected by center of pressure (CoP) displacements, while the participant remains stationary on a force platform [70, 71]. Metrics, such as the projection of the center of pressure on the anteroposterior axis (CoP-Y), have been utilized to study action tendencies objectively and are particularly suitable for assessing spontaneous preparation for approach-avoidance behaviors [72]. Specifically, a forward displacement of the CoP-Y is associated with approach tendency, whereas a backward displacement is associated with avoidance tendency [69].

Despite the potential of posturography to elucidate the interplay between emotion and postural control, the variability in findings has made it difficult to draw definitive conclusions. For example, studies of postural control during static standing have yielded conflicting findings. Gea et al. (2014) [73] found that approach behaviors were reflected by forward displacements of the CoP-Y axis when participants were exposed to dynamic facial expressions of happiness or pain. In contrast, Lebert et al. (2020) [32] reported no such effects when utilizing a range of static emotional faces and videos. Other authors have reported a reduction in the CoP displacements amplitude when exposed to unpleasant pictures, indicating a potential freezing response characterized by body immobilization [74, 75]. Additionally, in tasks where participants are required to initiate a step at a stimulus, faster reaction times have been observed for pleasant as compared to unpleasant visual stimuli [66, 76]. Furthermore, Mirabella et al. (2022) [30] reported a similar outcome when taking into account the arousal factor, observing no effect of it on the obtained results. Note that some other studies suggest that differences in arousal are stronger predictors of postural change than differences in valence [67, 77].

Despite the conflicting evidence, potentially due to factors such as arousal level and task relevance, measures like COP-Y displacements and step initiations continue to serve as valuable indicators of action tendencies by either decreasing (i.e., approach) or increasing (i.e., avoidance) the distance between the emotional stimulus location and the self [72, 78, 79]. Thus, it would be worthwhile to investigate how simulated changes in interpersonal distance, using approaching and withdrawing faces, may affect postural control. The introduction of dynamic facial stimuli could address the methodological limitations seen in prior studies, which have mainly used static images, thereby enhancing ecological validity. This choice is pivotal given that dynamic changes in facial position are more the rule than the exception in real-world interactions. To the best of our knowledge, this question, which has implications for both the theoretical domains of approach-avoidance action tendencies and interpersonal distance preference, is yet to be investigated.

Furthermore, while emotions can shape action tendencies, it has also been demonstrated that action tendencies can influence the categorization of emotional information. In a series of experiments involving emotional words, Neumann et al. (2000) [80] showed that passively perceiving or actively executing movements toward or away from the body can bias the categorization of positive and negative affective information, with a tendency to facilitate the categorization of positive emotions for approach and negative emotions for avoidance movements. In line with this, several studies have investigated how apparent approach and avoidance movements of emotional faces, simulating changes in interpersonal distance, affect participants’ perception of emotions. When participants were asked to categorize the emotion expressed by the face, approach movements facilitate the identification of happy faces, while avoidance movements facilitate the identification of angry faces [81, 82]. However, to our knowledge, no study has examined whether approach and avoidance tendencies of the observer can modulate interpersonal distance. This gap in the literature highlights the need for further investigation, as it could provide insight into a potential bidirectional relationship between action tendencies and interpersonal distance, similar to the established relationship between action tendencies and emotions [80, 83].

This study aimed to investigate the influence of emotional facial expressions on postural action tendencies and on interpersonal distance. Specifically, we seek to answer the following research questions:

1. Can emotional facial expressions, simulating approach or withdrawal movements, elicit observable postural changes?
2. How do emotional facial expressions influence preferred interpersonal distances?
3. To what extent do the action tendencies triggered by approaching or withdrawing emotional faces influence individuals’ preferred distance from others?

To achieve these goals, we conducted an experiment that involved studying the effects of emotional faces (happiness, sadness, fear, disgust, anger and neutral) simulating either an approaching or a withdrawing movements on two measures: (i) the spontaneous approach and avoidance behaviors through the postural parameter CoP-Y in a passive viewing task of the emotional faces, and (ii) the voluntary adjustment of preferred distances in response to emotional faces, using a computerized distance task.

In relation to our primary research question, we predicted that potentially affiliative or prosocial emotional expressions (e.g., happy, neutral, sad, fearful) would induce approach behaviors, whereas aversive emotional expressions (e.g., angry, disgusted) would induce avoidance behaviors. Furthermore, these tendencies are hypothesized to be modulated by the dynamic changes in faces movements. For example, an approaching happy face will amplify the approach tendency compared to a withdrawing happy face, and an approaching threatening face will amplify the avoidance tendency. Regarding our second research question, we hypothesized that participants would prefer greater interpersonal distance in response to aversive expressions like angry and disgusted faces, and shorter distances for affiliative expressions like happy, neutral, sad, and fearful faces. For our third research question, we aim to explore whether these action tendencies, as manifested through CoP-Y shifts, would influence the preferred interpersonal distances set by participants. We predict that approach or avoidance postural shifts will accentuate these preferred distances, making them either smaller or larger.

## Materials and methods

### Participants

The required number of participants was calculated using G*power 3.0 analysis [84]. Based on previous studies investigating the effects of emotion on posture (e.g., [55, 62, 85]), as well as on preferred interpersonal distance (e.g., [86, 87]), and considering the typical effect size of around 0.15 observed in this literature, we estimated the minimum number of participants required as 48 (f = 0.15, *α* = 0.05, and *β*” = 0.95). In all, seventy undergraduates completed the study from mars 2019 to february 2020, as a requirement for an introduction to psychology course. They were all native French speakers with normal or corrected vision. We also ensured that none of the participants had any neurological, psychiatric or significant depressive symptoms that can affect emotional processing [88, 89], or any postural issues such as scoliosis or recent surgery (see [90]). Based on individual data inspection, thirteen participants were not included in the following analysis: two for having stabilometric parameter values greater than three standard deviations beyond the group average and eleven for showing some loss of postural stability due to erratic movements such as self-touching or moving their lower limbs. The final analysis included data from fifty-seven participants (52 females; 5 males) who were analyzed (mean age = 20.2 ± 1.9 years old).

The study protocol was approved by the ethics committee from the Paris Descartes University (reference number n IRB: 20130500001072). All participants were informed about the procedure before the experiment and provided their written informed consent. Within 12 months of the data collection, the personal information gathered for this study has been pseudonymized and after 18 months it has been completely anonymized. True anonymization renders information non-personal.

### Stimuli

Sixty emotional faces were used as stimuli, sourced from the study by Lebert et al. (2021) [91]. The faces were computer-generated using FaceGen Modeller software (color front faces, hair removed, and direct gaze), and comprised ten identities (5 women, 5 men), each displaying six different facial expressions, namely happiness, fear, anger, sadness, disgust, and neutrality.

### Stimuli used for the computerized distance task

To create the computerized distance task and manipulate the perceived distance to each face, we followed the protocol used by Vieira et al. (2017) [44]. We generated faces of different sizes, ranging from the largest size (18.5 × 25 cm) simulating closer distance, to the smallest size (3.9 × 5 cm) simulating larger distance. The face sizes were incrementally reduced by 2.5 cm in height from the maximum size, resulting in a total of nine distinct face sizes. The adjustment in face size was calibrated to be noticeable yet subtle enough to avoid immediate detection by participants. In addition, we included a tenth size which corresponds to the dimension of a real face (14 × 18.5 cm), allowing for a more precise and realistic adjustment of distance in relation to others. This size was included to provide participants with an option for a typical distance of one meter from an interlocutor, as suggested by Hecht et al. (2019) [5].

To precisely control the perceived distance to each face in our computerized distance task, we calculated the visual angle for each face size using the formula below. Participants were placed at a fixed distance of 100 cm from the screen.

$$\begin{array}{c} \text{Visual}\;\text{angle}=2\times \text{atan}(\frac{\frac{\text{Stimuli}\;\text{size}}{2}}{\text{Stimulus}\;\text{distance}}) \end{array}$$

Using this visual angle, we were able to estimate the distance of a real face (18.5 cm) from the participant using the following formula:

$$\begin{array}{c} \text{Distance}\;\text{(cm)}=\frac{\frac{\text{18.5}\;\text{cm}}{2}}{\text{tan}(\frac{\text{Visual}\;\text{angle}}{2})} \end{array}$$

This allowed us to use simulated distances instead of image size or visual angles as a more explicit measure of perceived distance. For example, a real 18.5 cm face perceived at a visual angle of 14.25° would correspond to a distance of 74 cm from the participant, while a visual angle of 2.86° would correspond to a distance of 369 cm.

### Stimuli used for the postural task

To investigate postural responses to emotional faces that appear to approach or withdraw, participants underwent postural recording while passively viewing the emotional faces either increasing (approach) or decreasing (withdrawal) in size. Following the methodology used in previous postural studies [32, 91, 92], we generated 30-second image sequences, with the same facial stimuli used in the computerized interpersonal distance task. Each sequence included 10 distinct identities of the same emotion, presented for 3 seconds each. The identities were displayed either from smallest to largest (simulating an approaching movement) or from largest to smallest (simulating a withdrawing movement). There were 12 sequences of 30 seconds each, comprised of 2 stimulus movements (approach and withdrawal) for each of the 6 emotions.

### Material

Faces were displayed on a Dell screen with a resolution of 1920*1200 pixels, placed at a distance of 1 meter from each participant, and positioned at eye height. The postural task was performed using a force platform (AMTI: AccuSway+^®^) that enabled the recording of the anteroposterior (AP) displacements of the CoP, allowing for the calculation of the mean position of the CoP on the anteroposterior axis (CoP-Y, in cm). The mean CoP-Y provided information about the displacement toward or away from the stimuli, and could therefore be considered as an index of action tendencies. Data were collected at a frequency of 100 Hz. In the computerized distance-task, the adjustment of the preferred distance required a Microsoft SideWinder Plug and Play GamePad (USB) joystick.

### Procedure

**Fig 1:**
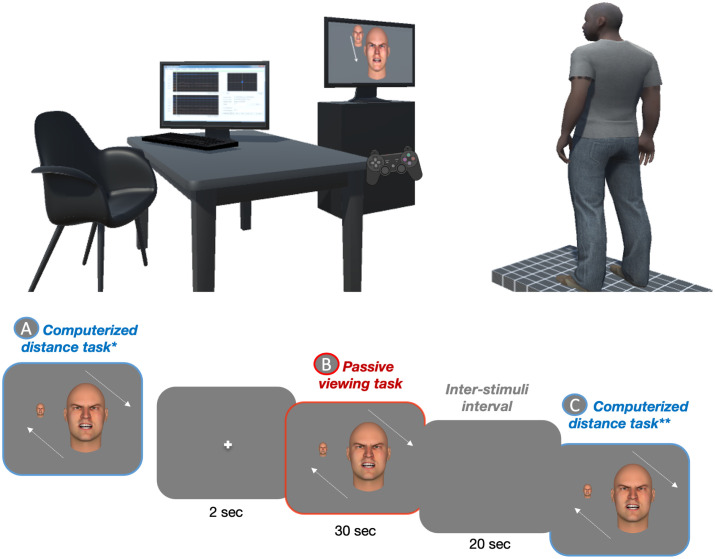
Schematic representation of the experimental tasks. The top panel of the graph illustrates the experimental setup where the participant stands on a force platform facing emotional faces that are either approaching or withdrawing. Participants took part in a three-part experiment consisting of: (A) an initial computerized stop-distance task, (B) a postural task where participants’ approach or avoidance tendencies were quantified using center of pressure (CoP-Y) displacements, and (C) a final computerized stop-distance task.

The experimental task was performed in a quiet room with a constant luminosity and was divided into two parts: i) an initial computerized distance task (without postural recording), ii) passive viewing of approaching or withdrawing emotional faces with postural recording followed by a computerized distance task (see Fig 1). The task was programmed and implemented using Opensesame [93].

#### Computerized distance task: Initial adjustment

Participants were placed on a force platform with their feet hip-width apart and their arms positioned along the body to maintain a comfortable posture. Next, participants performed the initial computerized distance task, which involved adjusting the preferred distance from emotional faces by changing their size on a computer screen using a joystick. The task consisted of 72 approach (faces were initially small, simulating a greater distance) and 72 withdrawal trials (faces were bigger, simulating a closer distance). Each included 6 identities repeated twice * 6 emotions. After a fixation cross of 600 ms, the face was displayed on the screen and participants used the joystick to increase (by pressing the “triangle button”) or decrease (by pressing the “cross button”) the face size until they felt comfortable with the distance for face-to-face interaction. They then locked their answer before moving on to the next trial.

#### Postural task followed by a computerized distance task

During the main experiment, participants completed twelve blocks, each comprising a postural task followed by a computerized distance task with the same emotion. Each block began with a fixation cross displayed on the screen for 2000 ms. Then, during the postural task, participants passively viewed a 30-sec sequence of emotional faces simulating an approach or withdrawal movement, while the displacements of their center of pressure (CoP-Y) were recorded. After each block, we ensured that participants had correctly identified the displayed emotions through verbal feedback, using a forced-choice task among the six basic emotions Next, participants performed the computerized distance task, which was similar to the initial adjustment task but only included the emotion presented in the previous passive viewing task. The stimuli were presented in a randomized order and the order of presentation of each block was counterbalanced between subjects.

### Statistical analysis

The primary statistical analyses consisted of examining whether emotional facial expressions combined with an approach or a withdrawal movement triggered action tendencies visible on the posture. The CoP-Y postural parameter was analyzed using 2 (faces movements: approach and withdrawal movement) x 6 (emotions: happy, fear, anger, sadness, disgust, and neutral expression) repeated measures analysis of variance (ANOVA). Planned comparisons were used for paired comparisons.

The secondary analyses entailed investigating whether the preferred distance was modulated by the expressed emotion of others and the action tendency induced by the perception of others’ movement. To do so, we compared the preferred distance before the triggering of action tendencies (initial adjustment) and after the approach or withdrawal postural blocks. We conducted a repeated measures analysis of variance (ANOVA) on the preferred distance assessed in the computerized distance-task using 3 (steps: initial adjustment, adjustment after approach postural blocks, adjustment after withdrawal postural blocks) x 6 (emotions: happy, fear, anger, sadness, disgust, and neutral expression) conditions. Planned comparisons were used for paired comparisons.

Finally, to deeper understand the association between spontaneous postural approach and avoidance tendencies and the preferred distance measured through the computerized distance task, we examined for each emotion the correlation between the CoP-Y and the subsequent preferred distance using Spearman correlation coefficients.

All the analyses were performed using R-statistical environment [94]. ANOVAs were computed using the “afex” package [95] followed by planned comparisons performed with the “emmeans” package [96]. Huynh-Feldt corrections were employed to adjust to the violation of the sphericity assumption in testing repeated measures effects. Bonferroni corrections were used to correct the p-values of multiple comparisons. A significance level of p = .05 was used for all statistical analyses. As a measure of the effect size, we calculated the partial eta-squared for the ANOVA and Cohen’s d for each planned comparison. Data are available on the OSF platform.

## Results

Postural data were baseline-corrected since the participants were liable to move on both the mediolateral and anteroposterior axes during the presentation of the initial fixation cross. All trials started from the same (0.0) coordinate at the beginning of the emotional stimuli presentation. It should be noted that the order of blocks presentation did not have any effect on the mean CoP-Y position (F(2,54) = 0.35, *p* >0.05), $\eta_{p}^{2}=0.013$ nor did they interact significantly with the stimuli movements (F(2,54) = 0.07, *p* >0.05), $\eta_{p}^{2}=0.003$ and the emotions (F(9.71, 262.22) = 1.34, *p* >0.05), $\eta_{p}^{2}=0.047$. Furthermore, the order of blocks presentation did not have any effect on the preferred distance (F(2,54) = 2.18, *p* >0.05, $\eta_{p}^{2}=0.075$) nor did they interact significantly with the stimuli movements (F(3.13, 84.41) = 1.93, *p* >0.05, $\eta_{p}^{2}=0.067$) and the emotions (F(3.08, 83.23) = 1.16, *p* >0.05, $\eta_{p}^{2}=0.041$).

**Fig 2:**
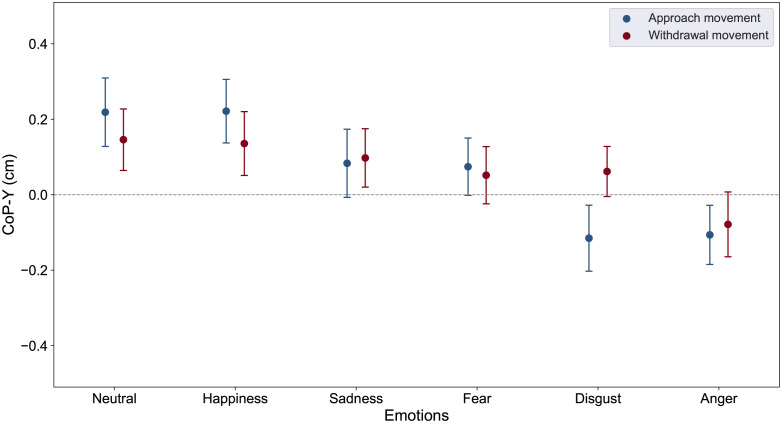
CoP-Y displacements. Mean COPY as function of Faces movements (approach postural blocks and withdrawal postural blocks) and Emotions (neutral, happiness, sadness, fear, disgust and anger). Error bars depict standard error of the means.

First, we conducted an ANOVA on the mean CoP-Y position to examine the effect of Emotions and Faces movements on approach-avoidance action tendencies. The ANOVA revealed a main effect of Emotions (F(5, 280) = 4.39, *p* <0.001, $\eta_{p}^{2}=0.073$). Planned comparisons (see Fig 2) showed that the mean CoP-Y was located significantly further forward in response to neutral and happy faces (M = 0.18, SE = 0.07 and M = 0.18, SE = 0.07 respectively) compared to fearful and sad faces (M = 0.06, SE = 0.05 and M = 0.09, SE = 0.06 respectively, *p* <.05, Cohen’s *d* = 0.24), itself significantly further forward compared to disgusted and angry faces (M = —0.03, SE = 0.07 and M = —0.09, SE = 0.06 respectively, p <0.05, Cohen’s *d* = 0.33). For each of these pairwise emotions (i.e., neutral/happy, sad/fear, disgust/anger), no significant difference was observed (all *p* >0.3). Although Fig 2 shows that approaching disgusted faces elicit a backward mean COP Y whereas withdrawing disgust faces elicit a forward mean COP Y, the ANOVA did not reveal any effect of Faces movements (F<1) nor any Faces movements * Emotions interaction (F(5,280) = 1.00, *p* = 0.41) on the mean CoP-Y.

Second, we investigated if the preferred distance adjusted before the action tendencies triggering (initial adjustment) differed from the one established after the action tendencies triggering (after approach and avoidance postural blocks).

The ANOVA revealed a main effect of the Steps (F(1.55, 86.78) = 17.39, *p* <.001, $\eta_{p}^{2}=0.24$), a main effect of the Emotions (F(1.52, 85.28) = 48.31, *p* <.001, $\eta_{p}^{2}=0.46$), but no Steps * Emotions interaction (F(4.60, 257.44) = 1.61, *p* = 0.16). Planned comparisons revealed that participants set greater distance during the initial adjustment (M = 151.86, SE = 5.99) than after postural blocks (M = 142.48, SE = 1.42, *p* <.001, Cohen’s *d* = 0.27). The preferred distances did not differ after the approach (M = 143.36, SE = 5.61) or withdrawal (M = 141.60, SE = 5.28, *p* >0.05) postural blocks.

**Fig 3:**
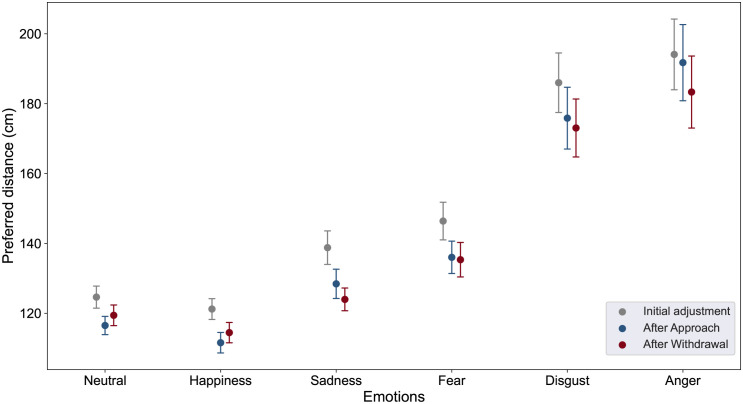
Preferred distance. Mean preferred distance as function of Steps (initial adjustment, adjustment after approach or withdrawal postural blocks and Emotions (neutral, happiness, sadness, fear, disgust and anger). Error bars depict standard error of the means.

Interestingly (see Fig 3), the preferred distances set in response to neutral (M = 120.19, SE = 2.38) and happy faces (M = 115.76, SE = 2.73) were significantly shorter than with fearful (M = 139.25, SE = 1.91) and sad faces (M = 130.40, SE = 1.85) (*p* <.001, Cohen’s *d* = 0.80), itself significantly shorter than the preferred distances to angry (M = 189.72, SE = 4.17) and disgusted faces (M = 178.30, SE = 3.38) (*p* <.001, Cohen’s *d* = 0.96). For each of these pairwise emotions, no significant difference was observed (all *p* >0.08).

**Fig 4:**
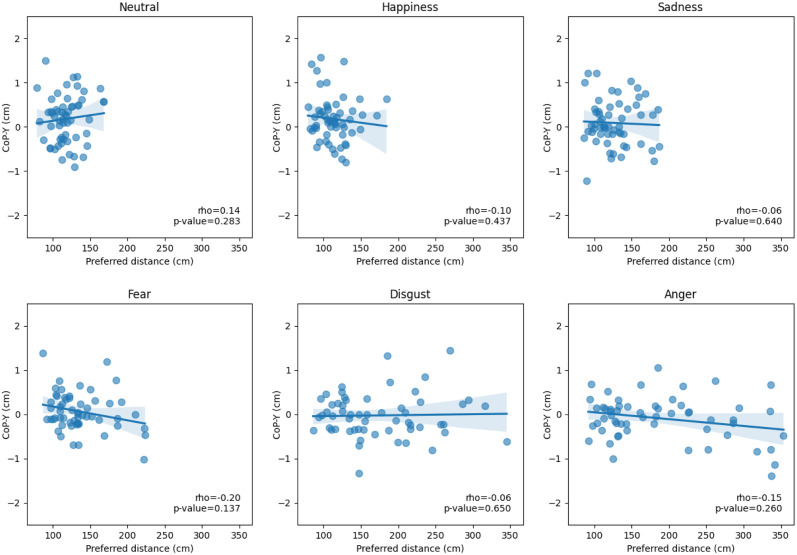
Correlations between the CoP-Y (in cm) and the preferred distance (in cm) for emotional faces after postural blocks. Spearman Rho and p value are provided for each emotional face.

Finally, we investigated whether there is an association between the mechanisms of spontaneous approach and avoidance tendencies, observe on posture, and the mechanisms of interpersonal distance regulation, voluntarily adjusted through a computerized distance task. For each emotion, we examined the correlation between the CoP-Y and the preferred distance. We did not observe any significant correlations between the CoP-Y and the preferred distance (see Fig 4) for emotional faces of neutral (*r* = 0.14, *p* = 0.28), happiness (*r* = -0.10, *p* = 0.44), fear (*r* = -0.20, *p* = 0.14), sadness (*r* = -0.06, *p* = 0.64), disgust (*r* = -0.06, *p* = 0.65) and anger (*r* = -0.15, *p* = 0.26).

## Discussion

This study aimed to deepen our understanding of how others’ emotions shape approach-avoidance postural behaviors and preferred interpersonal distance. Our primary objective was to investigate whether emotional faces, simulating approach, or withdrawal movements of others could elicit measurable postural shifts indicative of approach or avoidance tendencies. Consequently, we quantified postural changes through COP-Y displacements in response to approaching or withdrawing emotional faces. The secondary objective was to explore, for the first time, the extent to which action tendencies triggered by approaching or withdrawing emotional faces influence individuals’ preferred distance from others.

First, our study yielded significant postural results that contribute to the existing literature on the influence of emotional facial expressions on postural control. Our findings support the idea that approaching and withdrawing faces expressing neutrality and happiness lead to a stronger approach tendency than fearful and sad faces, while disgusted and angry faces elicit avoidance tendencies. These findings contrast with the motivational theory of emotions which predicts automatic approach and avoidance behaviors towards positive and negative stimuli respectively [23, 24]. Previous studies have already pointed out the importance of other factors than valence in the relationship between emotion and action tendencies, in particular the task-relevance emotional content of the stimuli [29, 30, 97], or their potential for future interaction [33, 35]. Extending this framework, our study combines both postural and interpersonal distance measures in a socially-relevant context. Indeed, participants were not only required to identify the emotion displayed but also to indicate their preferred distance for a face-to-face interaction. Our results indicate that the readiness for social interaction associated with a given emotion overcomes its valence in modulating action tendencies. Specifically, despite all being negatively valenced, fear and sadness elicited greater approach behavior, while anger triggered avoidance behavior.

In utilizing dynamic stimuli, our methodology yielded action tendencies observable in CoP-Y and also corroborated the findings of previous work [73], which found that dynamic emotional stimuli induced greater body sway amplitude in the anterior-posterior axis, thereby underscoring their efficacy in eliciting salient cues for action readiness. This observation stands in contrast with the weaker postural coupling reported in studies that employed static images or videos [32, 91, 98]. Additionally, contrary to our expectations, we observed that directionality of the face’s movement —whether approaching or withdrawing— did not yield differential postural responses. This absence of an effect could potentially be attributed to the complexity inherent in capturing the full spectrum of real-world social interactions within a single measure such as CoP-Y displacement. Notably, while our analysis did not indicate any significant interaction between facial movements and emotions, a trend emerged for faces displaying disgust, characterized by backward and forward CoP-Y displacements during approach and withdrawal, respectively. Consequently, our work underscores the imperative of incorporating dynamic emotional stimuli for a more accurate assessment of emotions’ impact on postural control, and provides a nuanced analysis of the observed effects associated with different emotional expressions. It’s worth noting that these action tendencies are relatively subtle and could be considered proxies for underlying motor intentions, rather than overt actions [83]. Consequently, by employing posturographic techniques, as opposed to traditional joystick tasks, we provide a more granular understanding of how such tendencies are manifested in the body. This methodological approach clarifies the complex relationship between emotional states and postural changes, thereby enhancing our understanding of how emotions shape social interactions.

Second, our results not only confirmed but also extended the findings of previous studies on interpersonal distance. Specifically, we found that distances chosen in response to neutral or happy faces were shorter than those chosen in response to fearful or sad expressions. This aligns with previous research, providing further support for the influence of emotional expression on interpersonal distance [18, 44]. Additionally, our study revealed that participants tend to choose larger distances in response to disgusted and angry facial expressions. This finding is consistent with observations from computerized and virtual distance tasks [18, 99], underscoring the perceived threats associated with these expressions. The choice of larger distances may be considered a protective mechanism, akin to maintaining a “safety buffer zone,” that has evolved to protect our integrity through defensive action [100, 101].

Furthermore, by incorporating blocks of approaching and withdrawing emotional faces, our investigation delved deeper into the mechanisms of interpersonal distance adjustment. Interestingly, we observed that the preferred distance were generally shorter following the approach or withdrawal postural blocks compared to the initial adjustment. However, the pattern of distance adjustment was similar across all three steps, suggesting that this difference may be due to habituation to the task or to facial expressions, and no difference was observed in the preferred distance adjustment after the approach and withdrawal postural blocks. Specifically, participants initially chose larger distances, reflecting a common tendency to maintain greater interpersonal space during initial encounters [53, 102]. However, as familiarity with the task and facial expressions increased, a significant shift in the preferred distance occurred, indicating a process of habituation that led to the recalibration of the preferred interpersonal distance. This habituation effect was observed consistently across both approach and withdrawal postural blocks, independent of the specific movements involved. These findings provide insights into the dynamic regulation of interpersonal distance and suggest the role of repeated exposure in shaping social interactions.

Our results indicate that emotional facial expressions and approach/withdrawal movements elicit visible action tendencies on posture, whereas subsequent voluntary adjustments of interpersonal distance are primarily influenced by emotional expressions rather than by facial movements. This emphasizes the intricate interplay between emotional facial expressions, approach/withdrawal movements, and visible action tendencies in posture. Furthermore, although we observed similar effects on both posture and preferred distance, such as forward lean and shorter preferred distance for neutral and happy expressions, and backward lean and larger preferred distance for disgusted and fearful expressions, we did not find significant correlations between these postural measures and the preferred distances chosen by participants. The lack of correlation between postural responses and preferred interpersonal distance could be interpreted as a dissociation between the underlying mechanisms of these two aspects of social interaction. Cognitive appraisal theories offer a plausible explanation for this dissociation (e.g., [103–105]). Specifically, automatic reactions such as postural adjustments to emotional facial expressions may be governed by rapid primary appraisals that quickly assess the immediate significance of the emotional cue, such as its potential threat or benefit. On the other hand, the determination of preferred interpersonal distance might be influenced by a more complex set of secondary appraisals, incorporating considerations such as comfort level or personal preferences, thus requiring additional time for decision-making and planning. These findings highlight the complex nature of the interaction between emotions, posture, and interpersonal distance, highlighting the need for more nuanced research to elucidate the underlying mechanisms. A limitation of our study is that we did not measure arousal levels of the emotional stimuli. This leaves open the question of how arousal may influence the approach-avoidance behaviors we observed [67, 77].

While our findings do not yield a direct correlation between postural action tendencies and preferred interpersonal distance, they do offer insights into how emotional cues can influence each of these variables independently. Although the data are preliminary, they open the door for future research, particularly in understanding potential clinical applications for disorders affecting emotional functioning. Indeed, our work aligns with existing literature that investigates the reciprocal relationships between emotions and posture across diverse populations, including those with neurodevelopmental pathologies where emotional and postural difficulties often coexist (for review, see [106]). We hope that our work serves as a starting point for more extensive studies that could eventually contribute to improved diagnostic and therapeutic strategies.

## Conclusion

In conclusion, this study sheds light on the intricate relationship between emotional cues, postural action tendencies, and the regulation of interpersonal distance, all of which are critical factors in successful social interactions. By examining a diverse range of emotional faces, we found that different emotional expressions significantly influenced postural action tendencies, with happy and neutral faces evoking a stronger approach tendency and disgusted and angry faces eliciting an avoidance tendency. Furthermore, our findings provide additional evidence of the modulation of interpersonal distance regulation in response to emotional cues, revealing distinct adjustments based on different emotional facial expressions, including shorter distances in response to neutral or happy faces, and larger distances in response to disgusted or angry expressions. However, the absence of a correlation between postural measures and preferred distance suggests a dissociation between the mechanisms underlying spontaneous approach and avoidance tendencies and those involved in the voluntary regulation of interpersonal distance. Overall, these findings provide a foundation for further research on the underlying mechanisms driving these processes and their implications for interpersonal communication and overall social well-being.
